# The Rheological and Fatigue Properties of Waste Acetate Fiber-Modified Bitumen

**DOI:** 10.3390/molecules30081784

**Published:** 2025-04-16

**Authors:** Cheng Cheng, Kai Yang, Jianwei Luo, Shu Yang, Yong Yan

**Affiliations:** 1 Yunnan Provincial Key Laboratory of Wood Adhesives and Glued Products, Southwest Forestry University, Kunming 650224, China; 15687200390@163.com (K.Y.); ljw17861123782@163.com (J.L.); 2 School of Civil Engineering, Southwest Forestry University, Kunming 650224, China; 3 Guizhou Highway Development Group Co., Ltd., Guiyang 563099, China; gztrys123@gmail.com; 4 Faculty of Civil Engineering and Mechanics, Kunming University of Science and Technology, Kunming 650500, China; yanyong@stu.kust.edu.cn

**Keywords:** acetate fiber, bitumen, rheological properties, fatigue properties, pseudo-strain energy

## Abstract

The rheological properties of fiber-reinforced binders are remarkable. The research on acetate fibers as reinforcing agents is scant. Acetate fibers exhibit more environmental benefits than lignocellulose and other fibers. In this study, acetate fibers were pretreated with anhydrous ethanol as the extractant to disperse the fibers uniformly in the bitumen and the high/medium-temperature fatigue properties of waste acetate fibers blended with binders were investigated. Infrared spectroscopy (FT-IR) tests showed that pretreatment was effective in removing plasticizers from CBs so that the fibers could be more uniformly dispersed in the binders. The roadworthiness and fatigue performance of the adhesives were tested based on frequency sweep (FS), multiple stress creep recovery (MSCR), and linear amplitude sweep (LAS) tests with different CB (cigarette butt) doping levels. Ultimately, CBs were added to effectively improve all aspects of bitumen performance, but this phenomenon was not enhanced with an increase in the amount of admixture—optimal covariance was 0.25%. Moreover, a further correlation analysis was performed for the three traditional predicted fatigue failure points. The best correlation was R^2^ = 0.98 for a 50% decrease in dynamic shear modulus, followed by R^2^ = 0.96 for peak stress–strain, and R^2^ = 0.88 for fatigue factor.

## 1. Introduction

Bituminous pavements possess an absolute advantage on highways due to their unique properties. However, for long-term service at high temperatures and heavy loads, bituminous pavements are prone to rutting, cracking, and other damage [1]. This imposes higher requirements on bituminous pavements. In recent years, a wide range of scientific research has been performed on this topic, with fibers, as high-performance additives with excellent physicochemical properties, being widely used in adhesives [2,3]. The fibers absorb the glue and the lighter components of the bond, thus increasing the embedding force at the adhesive–fiber interface. They can form a “three-dimensional skeleton” in the bitumen, which absorbs the applied stresses and shares the applied loads with the binder, improving the defects of stress concentration in the binder [4]. The incorporation of fibers has been shown to enable binders, as well as bituminous mixtures, to exhibit better overall properties, including rutting resistance, fatigue resistance, high-temperature resistance, and improved temperature-stress sensitivity [5,6,7].

Currently, the fibers that are widely added in adhesives are basalt, polyester, lignin, and carbon fibers [8,9]. Different types of fibers have unique advantages and, therefore, other mechanisms of fiber modification and differences in modification properties. For example, the self-healing properties of basalt fibers improve the toughness as well as the dynamic viscoelastic properties of the adhesive [10,11]. Metal and glass fibers improve the rutting resistance of the adhesive properties and the effective retarding of crack extension [12,13]. Liu and Xia et al. found that the mechanical properties of lignin fiber bitumen mixes were superior to those of bamboo fiber bitumen mixes. However, bamboo fiber bitumen mixes were preferred over lignin fibers for their high-temperature stability, low-temperature crack resistance, and moisture stability [14,15]. Wu et al. reported that acetate fibers could enhance bitumen mixes’ deformation and high-temperature rutting resistance [16]. Acetate fiber mixes showed a better low-temperature crack resistance than other chemical-fiber-modified bitumen mixes and had a similar fatigue resistance [17]. Moreover, acetate fibers are more environmentally friendly than lignocellulose. Mohajerani et al. [18] added discarded cigarette filters to clay bricks to counteract the energy generated during the transportation of substrate bitumen. Abbas Mohajerani et al. [19,20] used the properties of acetate fiber, such as being porous and lightweight, to combine paraffin-wrapped butts with bitumen mixes, which reduced the density of the mixtures and reduced the heat transfer. Md Tareq Rahman [21] applied waste cigarette butts as cellulose fibers in bitumen concrete and utilized their physical properties, significantly improving their rheological properties. However, all of the above applications used acetate fibers that contained nicotine, tar, and other toxins, which specifically impact the environment. Thus, more research is needed on new acetate-fiber-modified binders. The plasticizer glycerol triacetate (GTA) in the new acetate fibers affects the uniform dispersion of the binder. Therefore, in this study, we chose the more environmentally friendly acetate fibers as reinforcing agents to be mixed with the binder. The pretreatment was considered to remove the GTA and to enhance the thermal stability property of the fibers, which was evaluated using FT-IR. The linear viscoelastic rheological property was investigated using the frequency sweep (FS) test, the fatigue property was evaluated using the linear amplitude sweep (LAS) test combined with the viscoelastic continuum damage (S-VECD) theory, the rutting resistance was evaluated using the multiple stress recovery (MSCR) test, and the viscoelastic composition of the acetate-fiber-modified bitumen was investigated using the Burgers Model.

## 2. Results and Discussion

### 2.1. Frequency Sweep Test

**Figure 1:**
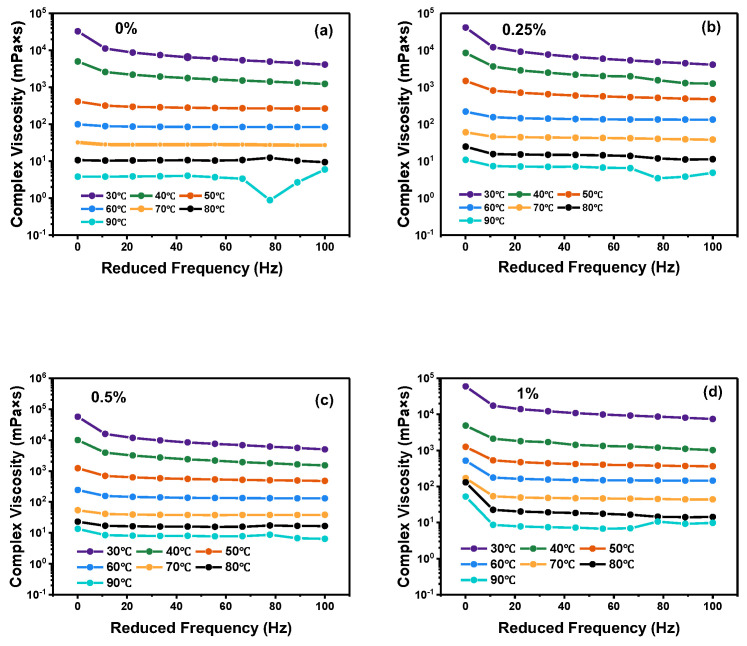
Self-healing of different CB-modified binders. (**a**–**d**) shows the frequency sweep curve of different content of CB fiber modified bitumen.

The variation in the complex viscosity of the adhesive at different frequencies is shown in Figure 1. The complex viscosity of the CB-modified binder increased more significantly with the addition of fibers. Macroscopically, the addition of fibers impeded the bitumen movement, and the fibers adsorbed the oil in the bitumen, resulting in a significant increase in the complex viscosity. As the frequency increased, the thickness of the binder tended to decrease, and the viscosity of the bitumen was less correlated with frequency around 40 °C, resulting in a viscosity curve that became almost a straight line, indicating that the bitumen conformed to the properties of a Newtonian fluid [22].

The viscosity coefficients of CB-fiber-modified bitumen at different admixtures are provided in Table 1. The fitting results show that the viscosity coefficient of the binder tended to increase gradually with the increase in temperature, indicating that the viscous flow state of the binder tended to be similar to that of a Newtonian fluid at high temperatures.

**Figure 2:**
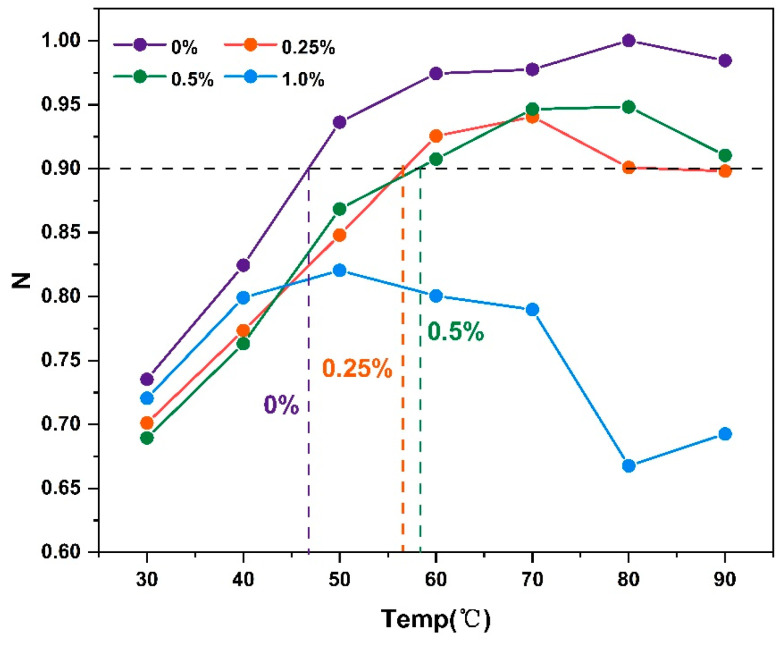
Flow index n at different temperatures.

From the results in Table 1 and the annual viscosity coefficient–temperature trend in Figure 2, it can be seen that the initial self-healing temperature increased with the addition of CB fibers, indicating that the addition of fibers reduced the fluidity of the CB-modified bitumen, which needed to be maintained at a higher temperature to achieve the same flow state. Notably, the viscosity coefficient of the binder did not reach 0.9 when the fiber admixture was at 1.0%, indicating that the fluidity of the bitumen mastic was seriously hindered at higher fiber admixtures, making it difficult for the binder to reach its initial self-healing temperature, making it more prone to cracking, etc.

### 2.2. Multiple Stress Creep Recovery Test

**Figure 3:**
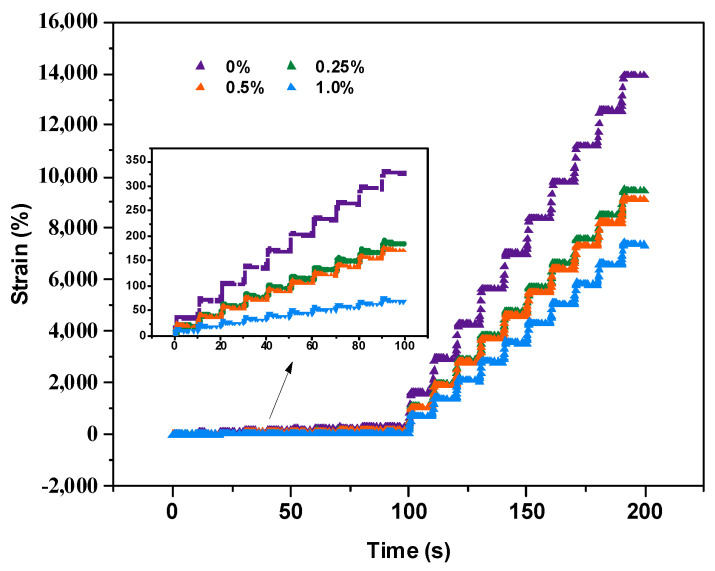
Stress–strain for different levels of CB doping.

To further analyze the high-temperature performance of CB fibers and binders, multi-stress repeated creep tests were conducted at 58 °C for bitumen modified with different levels of CB doping. Figure 3 shows the curves of the time–strain response of other adhesives at 58 °C, including the cumulative strain values for the first 100 s under 0.1 kPa stress versus the last 100 s under 3.2 kPa stress. The cumulative strain value of the binder with the addition of CB fibers decreased continuously with the increase in the dosing, showing a significant decreasing trend and a good positive effect on the high-temperature performance of the base bitumen.

Notably, the increase in the 0.5% CB-modified binder was almost negligible compared to that in the 0.25% CB-modified binder; the positive effect of CB-fiber modification was limited, and high quantities of fiber admixtures affected the performance of the binder. This was because, when the fiber content exceeded 1.0%, it became “dominant” in the binder. From the point of view of “polymer long chains” [23], the disorderly distribution of fibers was able to absorb most of the disordered distribution of fibers and concentrated stresses, allowing the bitumen to remain ductile, resulting in less deformation.

**Figure 4:**
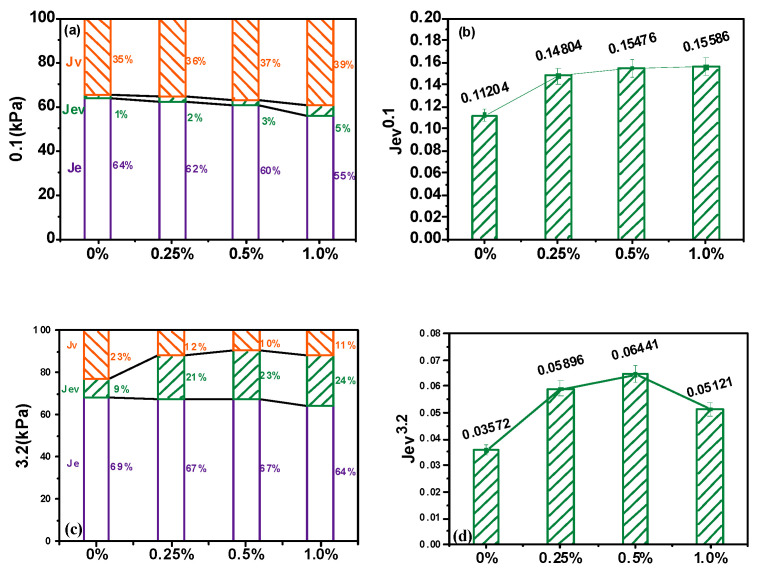
Viscoelastic components at 0.1 kPa (**a**) and 3.2 kPa (**c**); delayed elasticity at 0.1 kPa (**b**) and 3.2 kPa (**d**).

Bitumen behavior can be divided into three parts during creep loading: instantaneous elasticity Jv, delayed elasticity Jev, and viscous deformation Je. The four-parameter Burgers’ principal equation separates and calculates the three parts. Further analysis was conducted on the effect of different stresses and CB fiber doping on their components. From the results shown in Figure 4a,c, it is evident that both the binder and modified binder were dominated by the elastic component, under both 0.1 kpa or 3.2 kpa stresses. Furthermore, the addition of CB fibers led to the reduction in the flexible part of the binder, and this flexible part gradually hardened with the increase in doping, and the vicious and viscoelastic amounts increased with the rise in doping, especially of the viscoelastic component. This was because CBs have well-developed fiber voids that absorb the lighter members of the adhesive, thereby causing the bond to become sticky. However, Jev, under different stresses, showed a substantial decrease in its self-healing capacity, indicating that heavy loads and high strains were the leading extrinsic causes of irrecoverable adhesive deformation.

With the incorporation of CB fibers, as seen in Figure 4b,c, the deformation recovery of the adhesive improved. These fibers were the skeleton of the binder that could absorb most of the external forces, thus improving the ability of the binder to resist external pressures. However, the increase in Jve was no longer significant after exceeding 0.25%, and a decrease occurred at 0.1 Kpa, indicating an optimal amount of fiber incorporation.

### 2.3. Damage Tolerance Assessment Using Linear Amplitude Sweep

**Figure 5:**
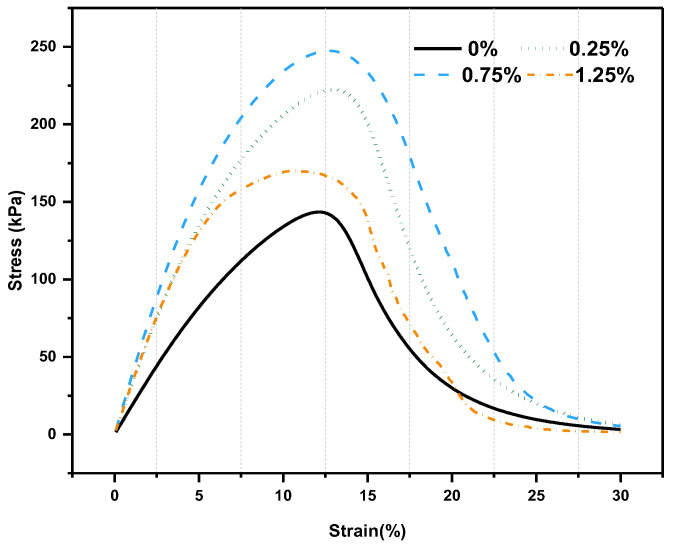
LAS-based stress–strain curves.

Figure 5 shows the strain–stress variation curves of different adhesives in the LAS test, which were used to investigate the fatigue damage characteristics of bonds. The trend of change was similar for each cement. Initially, the stress increased with increasing strain, and after reaching the peak strain, the stress decreased continuously as the strain continued to grow.

The different stress peaks can be considered as an effect of material stiffness. CB-modified adhesives have higher stress peaks and wider strain intervals than matrix adhesives, and they vary with the amount of admixture. After all, the fibers in the adhesive act as reinforcement, and the increased elasticity of the adhesive leads to a broader deformation interval because the fibers increase the toughness and can withstand more complex applied stresses. However, noteworthily, the peak strain of the binder appeared to decrease gradually with the addition of fibers when the doping level exceeded 0.5%, and the data points were not continuous when the fiber doping level reached 1.0%, indicating that more fibers caused the binder to harden and the fatigue life to reduce.

**Figure 6:**
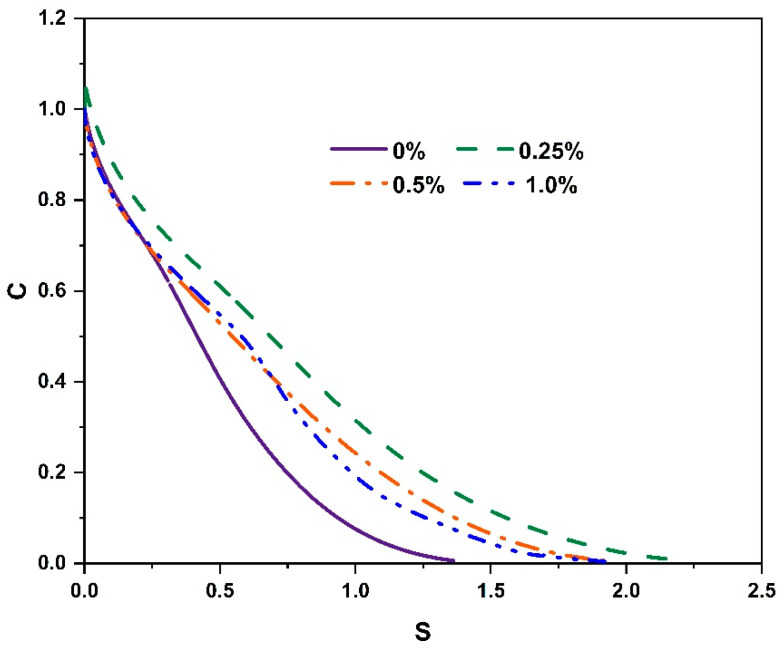
S-VECD-based C-S diagram.

The LAS results can be based on the S-VECD damage model of PES to predict the lifetime of different adhesives, as shown in Figure 6. The four adhesives showed a sharp decrease in the material integrity parameter C, followed by a slight decrease, as the cumulative damage parameter D increased. Evidently, for the same D, the order of C was 0.25%, 0.5%, 1.0%, and 0%, indicating that the incorporation of CBs into the adhesives effectively improved the damage resistance; the same pattern was observed when the material was wholly damaged (C = 0) for the same C.

**Figure 7:**
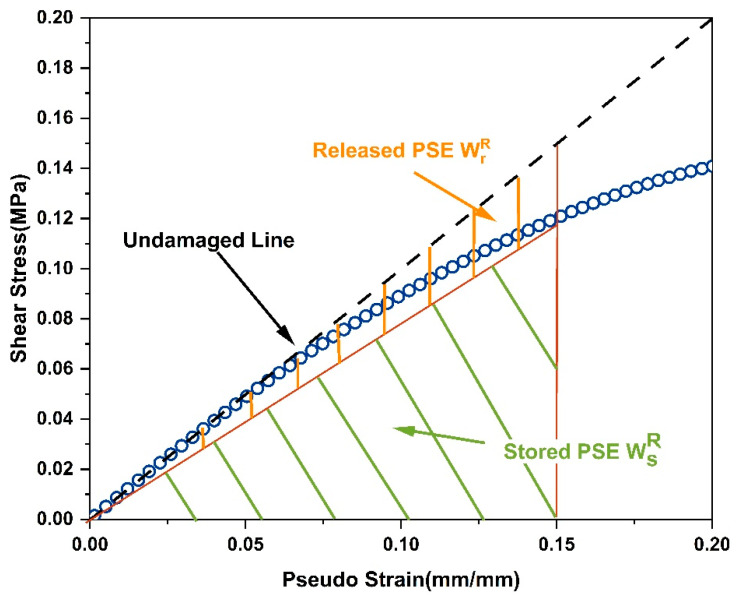
Pseudo-strain energy schematic.

In order to define fatigue failure indicators that can be applied in the S-VECD model, Wang et al. analyzed the LAS experimental data in the imaginary strain $\gamma^{r}$ coordinate system, which can eliminate the effect of material viscoelasticity and thus allow for a separate quantitative analysis of the damaging effect caused by the load [24]. Figure 7 shows the research conducted on the imaginary strain coordinates. At the beginning of the test, the material was in a non-damaged state and stored all the energy transferred by the applied load; however, as the external force of the load increased, the strain level of the material gradually increased, resulting in a deviation from the reference line of the non-damaged state. The material itself had a limited ability to store energy; thus, it released energy outside, causing damage to the material, and when the peak of stored energy was reached, it was defined as the failure point of material fatigue damage.

The maximum pseudo strain energy (PSE) input to the material ($W_{S}^{R}$) can be calculated according to Equation (6).

(1) $$W_{S}^{R}=\frac{1}{2}\times \tau_{P}\times \frac{\gamma_{P}^{R}}{DMR}=\frac{1}{2}\times C\times {\left(\gamma_{P}^{R}\right)}^{2}$$

The total PSE of the input material ($W_{t}^{R}$) is calculated from Equation (7).

(2) $$\int_{V}W_{t}^{R}dV=\int_{V}\frac{1}{2}\times \tau_{u}\times \gamma_{P}^{R}dV=\int_{V}\frac{1}{2}\times {\left(\gamma_{P}^{R}\right)}^{2}dV$$

Then, the PES ($W_{r}^{R}$) released by the material due to damage is

(3) $$W_{r}^{R}=W_{t}^{R}-W_{S}^{R}=\frac{1}{2}\times \left(1-C\right)\times {\left(\gamma_{P}^{R}\right)}^{2}$$

**Figure 8:**
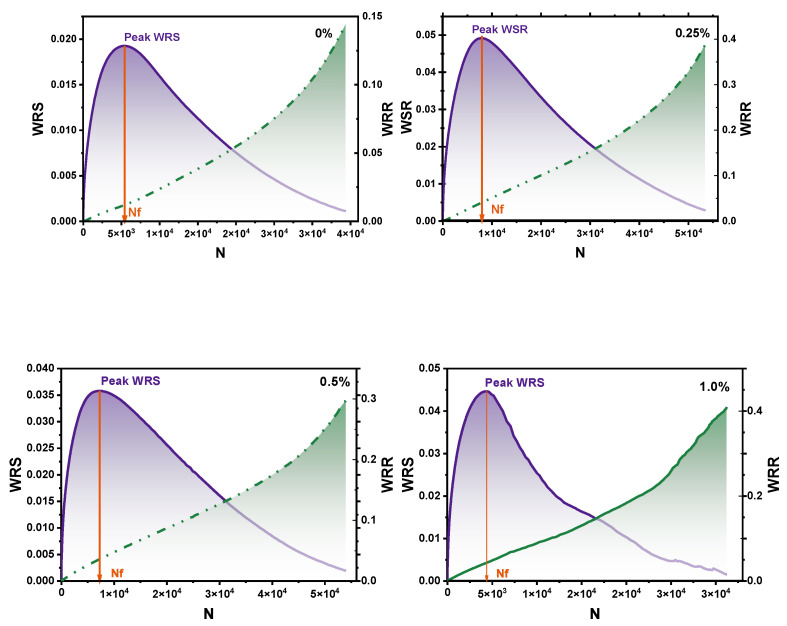
Pseudo-strain energy of binders at different CBs.

The analytical plots of bitumen modified with different CB fibers in imaginary strain coordinates are shown in Figure 8. Overall, each binder was undamaged at the beginning of the test, and as the number of cycles increased and stress was loaded, the storage energy WSR kept increasing and decreasing after reaching the peak of WRS. In contrast, the released energy WRR increased with the number of loadings due to the continuous damage to the material and, thus, the constant release of energy.

**Figure 9:**
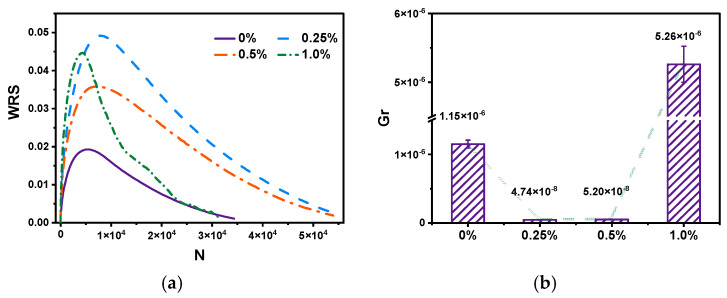
Pseudo-strain energy of bitumen (**a**); energy release rate Gr of bitumen (**b**).

The addition of fibers can effectively improve the number of cycles of the binder, as shown by the increase in fiber doping, and this effect becomes increasingly apparent in Figure 9a. From the colloid theory point of view, the viscosity increased, but a three-dimensional structure also formed in the binder, and the binder shared the applied stress to increase the number of cycles. However, notably, after the fiber doping exceeded 1.0%, the processes were reduced by 1/3. The excessive fibers changed the binder’s phase state, which was more likely to produce fatigue, indicating that more fiber doping did not produce better effects.

Furthermore, by analyzing the imaginary strain coordinate system, all four CB-modified binders had WSR peaks, which was the onset of the fatigue failure of bitumen. With the increase in fiber doping, the storage energy WSR and $N_{f}$ showed a regular pattern of an initial increase and then a decrease. The storage energy WSR of 0.25% CB-modified binder was more than twice that of the matrix bitumen, which could resist more substantial energy, while the WSR of 0.5% CB-modified bitumen decreased compared with that of 0.25% CB-modified bitumen.

(4) $$G^{R}=\frac{\bar{w_{r}^{R}}}{N_{f}}=\frac{A}{{\left(N_{f}\right)}^{2}}$$

where $N_{f}$ is the number of cycles *N* under the peak of $W_{S}^{R}$ and *A* is the integrated area of the $W_{r}^{R}$ curve reaching $N_{f}$.

The results of the Gr energy release rate calculated by Equation (9) are shown in Figure 9b. A more prominent Gr indicates a faster energy release rate and speedier damage to the adhesive [25]. The 1.0% CB-modified fibers had the highest energy release rate Gr, followed by the matrix bitumen, while the 0.25% and 0.5% CB fiber binders had an equal Gr, with the 0.25% CB fiber binder having the lowest Gr. The addition of CB fibers was effective in slowing down the release of energy and thus improving the damage level of the adhesive, but the addition of CB fibers produced a plateau area, after which there were too many fibers in the adhesive, resulting in the adhesive between the fibers becoming weak and more prone to cracking and damage.

### 2.4. Comparison of Fatigue Point Correlations

**Figure 10:**
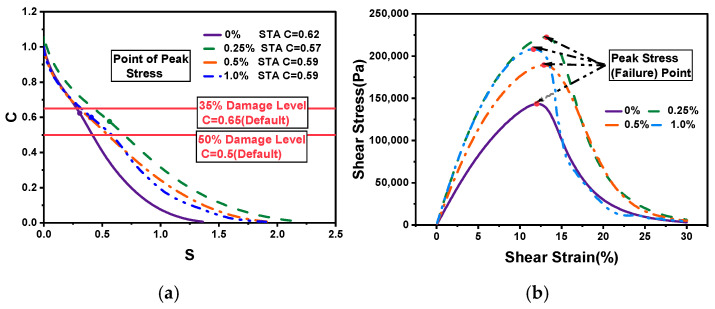
The corresponding C at different failure points (**a**); peak strain failure points (**b**).

Bitumen has an important influence on the fatigue properties of bituminous mixes; thus, the fatigue properties of bitumen have been a hot research topic [25]. In a previous study, Ref. [26] unified the failure criterion utilizing the peak virtual strain and the virtual strain release rate [27]. The peak stress was the yield stress of the adhesive, as shown in Figure 10, while the imaginary peak strain was after the yield stress of the adhesive.

Theoretically, the material should yield before failure. Thus, it was more reasonable to use the imaginary peak strain as the failure point of the adhesive.

**Figure 11:**
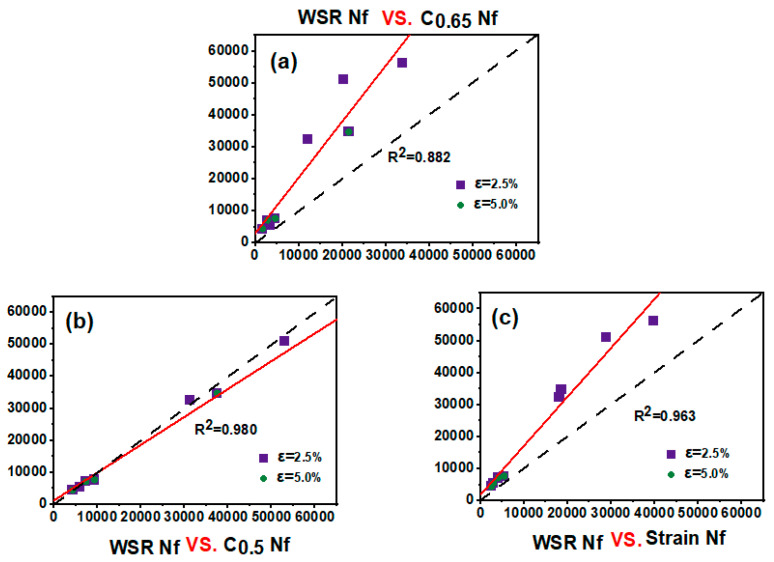
$N_{f}$ at C = 0.65 vs. $N_{f}$ at pseudo-strain energy (**a**); $N_{f}$ at C = 0. 5 vs. $N_{f}$ at pseudo-strain energy (**b**); $N_{f}$ at peak stress vs. $N_{f}$ at pseudo-strain energy (**c**).

Determining the bitumen fatigue failure point was particularly important for adhesive life prediction. The conventional method uses a 50% decrease in modulus (C = 0.5) as the fatigue failure point, while AASHTO TP 101-12 (American Association of State Highway and Transportation Officials, Washington, DC, USA) uses the initial modulus decay at 35% (C = 0.65) as the fatigue failure point and AASHTO TP 101-14 uses the C value corresponding to the peak stress or peak strain point at the peak stress–strain point as the fatigue failure point, which can be obtained from Figure 11b. The C value of strain at C = 0.5 had a lower strain level compared with C = 0.65 with the peak stress. Hence, using C = ?, which can be more reasonable and accurate, becomes an issue.

Therefore, the fatigue lives of the three failure points were calculated at different strains (2.5% and 5.0%) and compared with the WSR peak $N_{f}$ correlation, as shown in Figure 11, where the correlations for C = 0.5 and C = 0.65 were R^2^ = 0.980 and R^2^ = 0.963, respectively, both of which had a high correlation with WSR $N_{f}$, while the correlation of C = 0.65 was only R^2^ = 0.882, which was a poor correlation and a more conservative prediction. The 50% decrease in modulus was more consistent with $N_{f}$ peak, and theoretically, the fatigue failure point defined by C = 0.5 occurred after the yielding end of the material, which was consistent with the law that yielding occurs before the failure of the material. With this information and the three defined failure points, a 50% decrease in modulus predicted more reasonable failure points for adhesives.

## 3. Materials and Methods

### 3.1. Raw Materials

**Figure 12:**
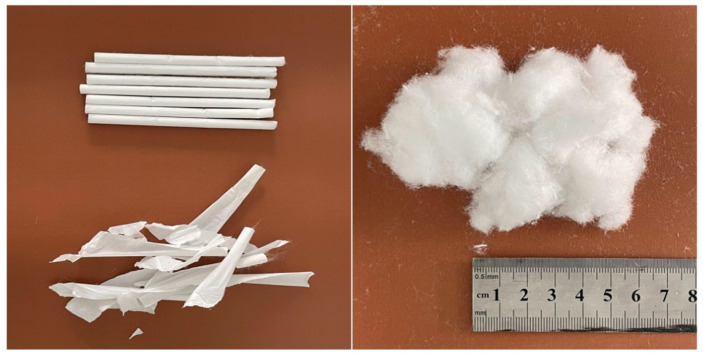
Appearance of acetate fibers.

The bitumen was 70# raw bitumen produced by SK Group of Korea and supplied by Yunnan Bitumen Reserve and Guarantee Center of China. The technical indexes met the technical requirements of bitumen of “Technical Specification for Highway Bituminous Pavement Construction” (JTG F40-2004). The technical indexes of base bitumen are shown in Table 2. This study used cigarette filters provided by Dali Prefecture, Yunnan Province Tobacco Company, as shown in Figure 12.

### 3.2. Fiber Preparation and Treatment

**Figure 13:**
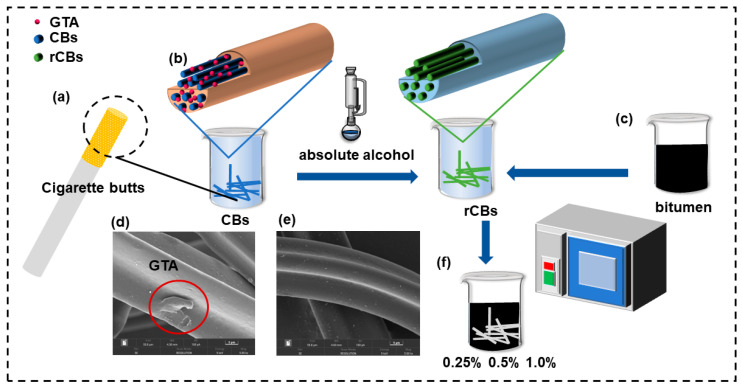
Cigarette structure (**a**); pretreatment of acetate fibers (**b**); bitumen heating (**c**); CBs (**d**); rCBs (**e**); preparation of CB fiber-modified binder (**f**).

The recycled acetate fibers were removed from the outer surface paper and cut with scissors to the specified length (≤6 mm). The recycled acetate fibers contained GTA with a 6–10% mass fraction. According to the gel theory, plasticizers disrupt polymer–polymer interactions, such as hydrogen bonding and van der Waals forces, with the migration of glycerol triacetate [28], increasing the inter- and intramolecular interaction forces between fibers, thus making it difficult to disperse the molded filter rods into flocs, which tend to gather into clumps between binders, affecting the performance of the fiber-modified bitumen. It was found that GTA could be precipitated in a crystalline state by extraction with acetate as the raw material and anhydrous ethanol as the extractant through a Soxhlet extractor [29]. Therefore, this study used ethanol as the extractant to treat acetate fibers. The process of cigarette filter treatment is shown in Figure 13. First, 450 g of base bitumen are heated at 90 °C in a furnace and held for 2 h to remove moisture. Then, the treated fiber was added to the matrix bitumen in a certain proportion (0.25%, 0.5%, and 1% by wt. of bitumen).

### 3.3. Pretreatment Evaluation

**Figure 14:**
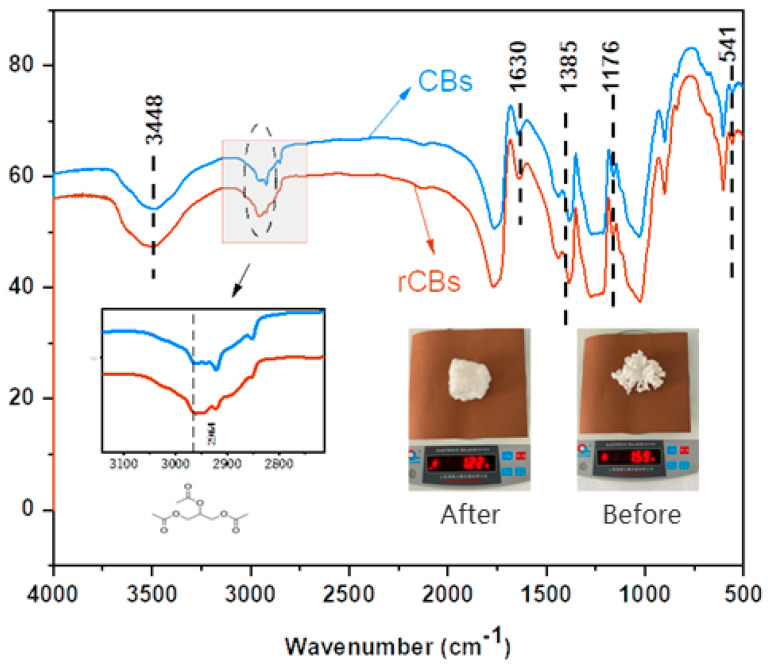
FT-IR and mass before and after pretreatment of acetate fibers.

The change in the mass of cigarette butts before and after pretreatment is demonstrated in Figure 14. The materials in the original sample weighed 1.59 g when extracted through the Soxhlet extractor. The churning and drying of the cigarette mass did not yield 1.22 g, and the original cigarette mass difference of 0.37 g accounted for about 23.2% of the mass. In the production of cigarette filters, in addition to cellulose acetate, the raw material contains a 10% mass fraction of GTA, 6–8% water, 0.2% titanium dioxide, and 1% oil agent [30]. Regarding the difference in mass, there was a partial loss of mass during the churning process, and the group was removed. Drying the sample in addition to pretreating it to remove moisture may remove the plasticizer GAT. In this study, an infrared spectrometer, Thermo Fisher Nioleti N10 (Waltham, MA, USA), was used to evaluate the effect of fiber treatment.

The functional groups in CB and rCB powders were characterized using infrared spectra, as shown in Figure 14. The two samples exhibited similar IR spectrograms, where the peaks appearing at a wave number of 3448 cm^−1^ corresponded to the stretching vibration of the O–H bond, while the mountains at wave numbers of 1385 cm^−1^ and 541 cm^−1^ corresponded to the bending vibration of the O-H bond [31]. The signal peaks at wave numbers of 1630 cm^−1^ and 1080 cm^−1^ were attributed to C=O bonding and C-O stretching vibrations, respectively [32]. In addition, the stretching vibrational signal peaks at wave numbers of 1550 cm^−1^ and 1176 cm^−1^ were observed for the C=N bond and C-N bond [33]. The FT-IR analysis further confirmed that the anhydrous ethanol pretreatment did not change the chemical structure of the fibers, and they remained the same substance. Notably, at 2964 cm^−1^, two samples showed different stretching vibrations due to the plasticizer GTA in CBs and the asymmetric bending vibration of CH3 in GAT 23. It can be concluded that the pretreatment effect significantly impacted CBs and that GTA can effectively extracted from CBs.

### 3.4. Preparation of Modified Fibers

A 450 g quantity of matrix bitumen was heated in a vacuum oven at 90 °C and maintained at that temperature for 2 h to remove moisture. Then, the treated fibers were added to the matrix bitumen in specific ratios (0.25%, 0.5%, and 1.0%) based on the bitumen mass. The preparation of acetate fiber-modified bitumen with an acetate fiber dosing of 0.25%, 0.5%, or 1.0 was completed according to the distribution mixing method, as shown in Figure 13f.

### 3.5. Evaluation of Fiber Pretreatment Effect

FT-IR analysis using a Bruker Alpha spectrometer (Bruker Corporation, Billerica, MA, USA) was performed in the wave number range of 380–4000 cm^−1^ with total reflectance to evaluate the acetate fibers’ pretreatment attenuation. FT-IR spectroscopy was also used to pinpoint the functional groups in the samples.

### 3.6. Frequency Sweep Test

The bitumen was subjected to a frequency sweep based on a dynamic shear rheometer (DSR) with 5% strain control, where the frequency scan represents the effect of different shear angle frequencies on the bitumen rheology at a specific temperature. Frequency scanning was performed at specific temperatures of 30 °C, 40 °C, 50 °C, 60 °C, 70 °C, and 80 °C, in the frequency range of 0.1–100 Hz.

The self-healing properties of CB-modified binders were further analyzed using Equation (1). The frequency scan test allows us to obtain the flow characteristics index *n* for different CB fiber bitumen slurries for the retest viscosity versus frequency, and thus, we obtain the following equation:

(5) $$\eta *={m\times |\omega |}^{n-1}$$

where |ω| is the frequency, η* is the composite viscosity, and *m* and *n* are the fitting parameters; *n* is also called the flow characteristics index, according to which the initial self-healing temperature of the fiber bitumen slurry is analyzed to study its self-healing ability.

### 3.7. Multiple Stress Creep Recovery Test

In order to better evaluate the high-temperature performance of the acetate fiber-modified bitumen, a multi-stress repetitive creep recovery test based on the DSR with 1 s loading and 9 s unloading for 20 creep recovery processes was conducted. The high-temperature performance of bitumen was evaluated by recording the delayed elastic recovery deformation and irrecoverable deformation of road bitumen under the action of external forces.

**Figure 15:**
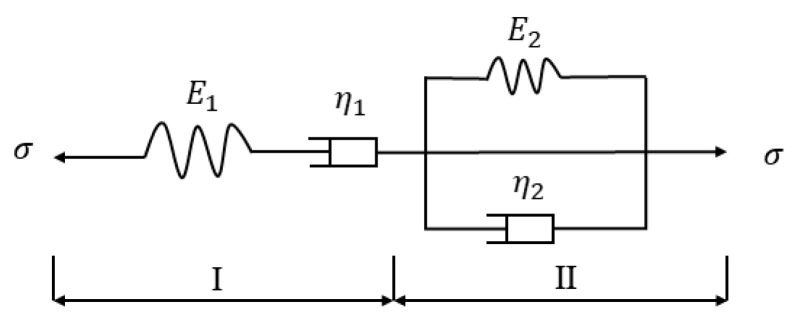
Burgers Model. I is max model and II is Kelvin model.

Bitumen usually transforms from a solid to a semi-solid at a sustained increase in temperature [26]. Under the loading effect of external forces, it exhibits the properties of a reliable semi-solid, i.e., viscosity and elasticity. In this process, the deformation of bitumen is not completely an elastic solid deformation or a viscous fluid deformation but is somewhere in between; thus, the Burgers Model can be used to characterize bitumen’s rheological properties [32]. The Burgers Model is a classical viscoelastic mechanic’s model, which is constituted by connecting the Maxwell Model in series with the Kelvin Model, as shown in Figure 15.

(6) $$J\left(t\right)=\frac{1}{E_{1}}+\frac{t}{\eta_{1}}+\frac{1}{E_{2}}\left(1-e^{-\frac{E_{2}}{\eta_{2}}t}\right)$$

where $J\left(t\right)$ is the creep flexibility at moment t, $Jv=\frac{1}{E_{1}}$ is the elastic deformation ratio of bitumen, $Jev=\frac{1}{E_{2}}\left(1-e^{-\frac{E_{2}}{\eta_{2}}t}\right)$ is the delayed elastic deformation ratio of bitumen, and $Je=\frac{t}{\eta_{1}}$ is the viscous deformation ratio of bitumen.

### 3.8. Damage Tolerance Assessment Using Linear Amplitude Sweep

These tests depended on the dynamic shear rheometer (DSR) apparatus and were performed at 25 °C with an 8 mm oscillating plate as the mold and a spacing of 2 mm for 310 s scans. The LAS test was divided into two steps. First, the frequency scan test was performed to determine the non-damaging material parameters by applying 0.1% strain to the adhesive in the range of 0.1–30 Hz at the beginning of the test. The second step involved a shear amplitude strain applied to the glue within the 0.1–30% shear strain range at 10 Hz.

(7) $$S\left(t\right)\approx \sum_{i=1}^{N}[{\frac{DMR}{2}\times {\left(\gamma^{R}\right)}^{2}\times (C_{j-1}-C_{j})]}^{\frac{\alpha }{1+\alpha }}\times {\left(t_{j}-t_{j-1}\right)}^{\frac{1}{1+\alpha }}$$

where DMR is the ratio of $G_{finger}$ to ${\left\lceil G*\right\rceil }_{0}$ (in order to eliminate the variability between parallel samples of the same material, DMR is generally taken as 0.9–1.1), and $\gamma^{R}$ is the required strain shear modulus of the loading cycle determined by Equation (4), where *C* is the pseudo-stiffness, determined by the Equation $C\left(S\right)=\frac{\tau_{P}}{\gamma_{P}^{R}\times DMR}$.

(8) $$W_{S}^{R}=\frac{1}{2}\times \tau_{P}\times \frac{\gamma_{P}^{R}}{DMR}=\frac{1}{2}\times C\times {\left(\gamma_{P}^{R}\right)}^{2}$$

The pseudo-modulus *C* of the material is further fitted to the damage variable *S* according to Equation (5):

(9) $$\left|G*\right|\sin\delta =C\left(j\right)=C_{0}-C_{1}{\left(D\right)}^{C_{2}}$$

where *C*_0_ is 1 and *C*_1_ and *C*_2_ are the best-fit parameters obtained from the planning solution.

## 4. Conclusions

A series of tests was carried out using the dynamic shear rheometer (DSR) and infrared spectroscopy (FT-IR) to characterize the surface characteristics of CBs before and after pretreatment compared with those of CBs in adhesives, and the conclusions drawn are as follows:

1. Using anhydrous ethanol as the extractant can effectively remove the plasticizer in CBs, which makes the fibers less likely to agglomerate during the mixing process with the binder, and this enhances the degree of uniform dispersion of fibers in bitumen. It also makes the fiber surface rougher and increases the embedding force of the binder.
2. The addition of fibers impedes the flow of the binder, thereby increasing the initial healing temperature and improving the rutting resistance of the binder. However, high quantities of added fibers can cause the binder to harden and become more prone to cracking.
3. Evaluation from the point of view of pseudo-strain: The addition of fibers can effectively improve the cracking resistance and increase the predicted fatigue life. When too many fibers are present, many fine cracks are produced, thus accelerating cracking and reducing the cracking resistance. Acetate fibers positively affect the binder, but more acetate fibers are not always better, as there is a “platform zone” beyond which performance decreases.
4. For an acetate fiber-modified adhesive, the fatigue-life failure point was compared using pseudo-strain up to 50% of conventional strain (C = 0.5), fatigue factor (C = 0.65), and peak stress in the stress–strain diagram, and the best prediction was obtained for the traditional strain up to 50% (C = 0.5), followed by peak stress, and the worst prediction was obtained for the fatigue factor.

## Figures and Tables

**Table 1:** Flow index *n* at different temperatures.

n	0%	0.25%	0.5%	1.0%
30 °C	0.735	0.701	0.689	0.720
40 °C	0.824	0.659	0.763	0.627
50 °C	0.936	0.848	0.868	0.820
60 °C	0.974	0.925	0.907	0.800
70 °C	0.977	0.940	0.946	0.789
80 °C	1.000	0.900	0.948	0.667
90 °C	0.984	0.898	0.910	0.692

**Table 2:** Technical specifications of raw bitumen.

Experimental Projects	Unit	Test Result	Technology Requirements
Needle penetration (25 °C, 5 s, 100 g)	0.1 mm	65.4	60–80
Softening point	°C	46.6	≥46
Ductility (15 °C)	cm	>150	≥100
TFOT needle penetration ratio (25 °C)	%	76.9	≥61%
Residual ductility (15 °C)	cm	132.9	≥15

## Data Availability

All data generated or analyzed during this study are included in this published article.
